# Sp1 acetylation is associated with loss of DNA binding at promoters associated with cell cycle arrest and cell death in a colon cell line

**DOI:** 10.1186/1476-4598-9-275

**Published:** 2010-10-15

**Authors:** Jennifer S Waby, Haridasan Chirakkal, ChenWei Yu, Gareth J Griffiths, Roderick SP Benson, Colin D Bingle, Bernard M Corfe

**Affiliations:** 1 Department of Oncology, University of Sheffield, Medical School, Beech Hill Road, Sheffield, S10 2RX, UK; 2 Department of Infection and Immunity, University of Sheffield, Medical School, Beech Hill Road, Sheffield, S10 2RX, UK; 3 Imagen Biotech Ltd, 48 Grafton Street, Manchester, M13 9XX, UK; 4 Kemin Industries, South Asia, Pvt.Ltd., The Trapezium, Second Floor No:39, Nelson Manickam Road, Chennai-600 029 Tamilnadu, India; 5 Current address: Department of Biological Sciences, University of Hull HU6 7RX UK

## Abstract

Butyrate, a known histone deacetylase inhibitor (HDACi) and product of fibre fermentation, is postulated to mediate the protective effect of dietary fibre against colon cancer. The transcription factor Sp1 is a target of acetylation and is known to be associated with class I HDACs, including HDAC1. Sp1 is a ubiquitous transcription factor and Sp1-regulated genes include those involved in cell cycle regulation, apoptosis and lipogenesis: all major pathways in cancer development. The only known acetylated residue of Sp1 is lysine703 which resides in the DNA binding domain. Here we show that acetylated Sp1 loses p21- and bak-promoter -binding function *in vitro*. Furthermore treatment with a panel of HDAC inhibitors showed clustering of activities for a subset of inhibitors, causing G2 cell cycle arrest, Sp1 acetylation, p21 and Bak over-expression, all with very similar EC_50 _concentrations. These HDACi activities were not distributed according to the molecular class of compound. In order to mimic loss of binding, an siRNA strategy was used to reduce Sp1 expression. This resulted in altered expression of multiple elements of the p53/p21 pathway. Taken together our data suggest a mechanistic model for the chemopreventive actions of butyrate in colon epithelial cells, and provide new insight into the differential activities some classes of HDAC inhibitors.

## Introduction

It is now widely recognised that histone acetyltransferases (HATs) and histone deacetylases (HDACs) have non-histone substrates and can modulate transcription by directly acetylating/deacetylating transcription factors and associated cofactors [1]. Two members of the Sp transcription factor family, Sp1 and Sp3, have been reported to be acetylated [2,3]. Alanine scanning mutagenesis identified lysine-703 (K703) as a target of acetylation in Sp1 [3]. Sp1 K703A mutants showed no detectable acetylation suggesting that acetylation only occurs at this single site. Sp3 has also been reported to be acetylated at a specific residue in its C-terminal inhibitory domain, however mutants lacking this domain were still acetylated, therefore Sp3 probably has other residues which are acetylated [2]. The functional relevance of this post-translational modification is unclear from the literature. The location of the Sp1 K703 acetylation site in the DNA binding domain suggests acetylation of Sp1 could affect DNA binding and/or gene transactivation. Initial findings indicated that acetylation may increase Sp-mediated transcription [4]. However, the simplistic 'more acetylation results in more transcription' model has been disputed by recent findings. Expression of a recombinant Sp1 mutant (K703A), which could not be acetylated, resulted in increased expression of the lipoxygenase (12-LOX) gene [3] whilst treatment with HDAC inhibitors attenuated the expression of COX-2 in HT29 cells and IGFBP3 in CaCo2 cells [5,6]. It is possible that competition between Sp1 and Sp3 for GC-box binding sites, could be swayed by acetylation. In support of this, Sp3, which is normally a weak transcriptional activator, was able to, in the absence of acetyltransferases, function as a transcriptional activator with similar potency to Sp1 [7]. Small alterations in Sp1/Sp3 binding affinity could result in altered occupancy at the promoter and alter the gene expression according to whether the resident transcription factor is an activator or repressor. Chromatin immunoprecipitation (ChIP) assays have demonstrated a reduction in binding of Sp1 accompanied by an increase in Sp3 binding at the major vault protein promoter following treatment with the HDAC inhibitors TSA and butyrate [8]. A similar switch of Sp1 for Sp3 has been observed at the promoter for the pro-apoptotic protein BAK following butyrate treatment [9].

Sp1 and Sp3 are reported to be associated with HDAC1 and HDAC2 [10,11]. HDAC1 may be present in a large multimeric complex with Sp1, Sp3 and p300 during IGFBP-3 activation [12]. HDAC1 also binds directly to Sp1 zinc fingers, however, this is a deacetylase activity-independent event [13]. Taken together these data support a role for HDAC1 and HDAC2 in the deactylation of Sp1 and Sp3. Overexpression of class I and II HDACs has been observed in a number of cancers including gastric [14], lung [15], breast[16], colon [17], and ovarian cancers [18]. These observations have led to interest in HDAC inhibitors as potential therapeutic targets, with emphasis on development of novel HDAC inhibitors (HDACi).

HDAC inhibitors have been studied for several years. Butyrate, a by-product of fibre fermentation in the colon was characterised as a promoter of histone acetylation in 1977 [19]. Several other naturally occurring HDACi have been identified, including trapoxin and valproic acid. There is a substantial academic and industrial effort to develop inhibitors with HDAC-specific activity. Several classes of compounds have HDACi activity, including short-chain fatty acids, such as butyrate, branched chain fatty acids, hydroxamic acids and others (see Additional File 1, Table 1). The different classes of inhibitors have variable and overlapping specificity for each HDAC, although with the exceptions of tubacin (HDAC 6 specific) and cambinol (Sirtuin class 1/2 specific), none are truly specific. Most HDACi act against several members of each subclass of HDACs, and several of the hydroxamic acids are active against all Class I and II HDACs. Likewise nicotinamide is a pan-specific inhibitor of the sirtuin HDAC subfamily. Several clinical trials, particularly for cancer therapy, are underway to examine therapeutic benefits of HDACi. The HDAC inhibitor Vorinostat (suberoylanilde hydroxamic acid, SAHA), has been approved for treatment of cutaneous T cell lymphoma [20]. In many of these trials the HDACi is used as a combination therapy with a first-line chemotherapeutic in order to improve efficacy. Trials are in progress for both short and branched chain fatty acids, hydroxamic acids and cyclic tetrapeptides [reviewed in [21] and [22]]. In addition its to pharmacological relevance, butyrate occurs naturally in the colon, at pharmacologically active concentrations (0.5-20 mM) as a by-product of fibre fermentation and is thought to be responsible for protection against colorectal cancer conferred by high fibre diet [23,24].

**Table 1:** List of genes in the p53-p21 pathway downregulated following Sp1 knockdown

Probe Set ID	Gene Title	Gene Symbol	48 hr post Sp1 knockdown		72 hr post Sp1 knockdown	
			
			Fold change	Regulation	Fold change	Regulation
1556990_at	PERP, TP53 apoptosis effector	PERP	2.87	down	2.46	down

211725_s_at	BH3 interacting domain death agonist	BID	1.78	down	1.85	down
227143_s_at	BH3 interacting domain death agonist	BID	1.64	down	1.70	down
204493_at	BH3 interacting domain death agonist	BID	1.82	down	-	No change

209644_x_at	cyclin-dependent kinase inhibitor 2A (p16)	CDKN2A (p14ARF)	1.53	down	1.51	down
207039_at	cyclin-dependent kinase inhibitor 2A (p16)	CDKN2A (p14ARF)	1.58	down	-	No change

224851_at	cyclin-dependent kinase 6	CDK6	1.40	down	-	No change

209305_s_at	growth arrest and DNA-damage-inducible, beta	GADD45B	1.31	down	-	No change

214710_s_at	cyclin B1	CCNB1	1.30	down	-	No change

204315_s_at	G-2 and S-phase expressed 1	GTSE1 (B99)	1.23	down	-	No change

218424_s_at	STEAP family member 3	STEAP3 (TSAP6)	1.22	down	-	No change

236814_at	Mdm4, transformed 3T3 cell double minute 4, p53 binding protein (mouse)	MDM4	1.73	down	-	No change
225740_x_at	Mdm4, transformed 3T3 cell double minute 4, p53 binding protein (mouse)	MDM4	-	No change	1.74	down

202763_at	caspase 3, apoptosis-related cysteine peptidase	CASP3	-	No change	1.29	down

**Table 2:** List of genes in the p53-p21 pathway upregulated following Sp1 knockdown

Probe Set ID	Gene Title	Gene Symbol	48 hr post Sp1 knockdown		72 hr post Sp1 knockdown	
			
			Fold change	Regulation	Fold change	Regulation
220403_s_at	p53-regulated apoptosis-inducing protein 1	P53AIP1	2.76	up	-	No change

202627_s_at	serpin peptidase inhibitor, clade E (nexin, plasminogen activator inhibitor type 1), member 1	SERPINE1 (PAI)	2.01	up	2.43	up

230330_at	protein phosphatase 1D magnesium-dependent, delta isoform	PPM1D (WIP-1)	1.86	up	-	No change
204566_at	protein phosphatase 1D magnesium-dependent, delta isoform	PPM1D (WIP-1)	1.72	up	1.52	up

202284_s_at	cyclin-dependent kinase inhibitor 1A (p21, Cip1)	CDKN1A	1.84	up	1.96	up
1555186_at	cyclin-dependent kinase inhibitor 1A (p21, Cip1)	CDKN1A	-	No change	1.25	up

203409_at	damage-specific DNA binding protein 2, 48kDa (p48 subunit)	DDB2	1.79	up	-	No change

204859_s_at	apoptotic peptidase activating factor 1	APAF1	1.76	up	-	No change

218346_s_at	sestrin 1	SESN1	1.74	up	1.56	up

204285_s_at	phorbol-12-myristate-13-acetate-induced protein 1	PMAIP1 (NOXA)	1.48	up	1.60	up
204286_s_at	phorbol-12-myristate-13-acetate-induced protein 1	PMAIP1 (NOXA)	-	No change	1.55	up

204781_s_at	Fas (TNF receptor superfamily, member 6)	FAS	1.73	up	1.59	up
215719_x_at	Fas (TNF receptor superfamily, member 6)	FAS	1.47	up	1.35	up
204780_s_at	Fas (TNF receptor superfamily, member 6)	FAS	1.43	up	1.45	up
216252_x_at	Fas (TNF receptor superfamily, member 6)	FAS	1.41	up	-	No change

208712_at	cyclin D1	CCND1	1.46	up	1.39	up

208711_s_at	cyclin D1	CCND1	1.34	up	-	No change

201700_at	cyclin D3	CCND3	1.42	up	-	No change

222986_s_at	scotin	SCOTIN	1.40	up	-	No change

242899_at	sestrin 3	sesn3	1.38	up	-	No change
241015_at	sestrin3	sesn3	-	No change	1.43	down

208796_s_at	cyclin G1	CCNG1	1.38	up	-	No change

229415_at	cytochrome c, somatic	CYCS	1.35	up	-	No change

211711_s_at	phosphatase and tensin homolog	PTEN	1.25	up	-	No change

238075_at	CHK1 Checkpoint homolog (*S.Pombe*)	CHK1	1.23	up	-	No change

202981_x_at	seven in absentia homolog 1 (Drosophila)	SIAH1	1.20	up	-	No change

209260_at	stratifin	SFN (14-3-3σ)	1.33	up	-	No change
33323_r_at	stratifin	SFN (14-3-3σ)	-	No change	1.43	up
33322_i_at	stratifin	SFN (14-3-3σ)	-	No change	1.35	up

213523_at	cyclin E1	CCNE1	-	No change	1.34	up

204855_at	serpin peptidase inhibitor, clade B (ovalbumin), member 5	SERPINB5 (Maspin)	-	No change	1.32	up

201110_s_at	thrombospondin 1	THBS1 (TSP1)	-	No change	1.32	up

211692_s_at	BCL2 binding component 3	BBC3 (PUMA)	-	No change	1.23	up

Despite widespread interest in the development of HDACi as therapeutics, their mechanism of action is not fully understood. Piecemeal reports using one or two HDACi in single cell lines indicate that most of these compounds will drive both cell cycle arrest and apoptosis *in vitro*. Work using isogenic colon cell lines has revealed that p21 is essential for normal cell cycle arrest following butyrate treatment and several publications show that this occurs via a p53 independent pathway [25]. Our work and that of others has shown that the pro-apoptotic protein Bak is up-regulated following butyrate treatment of colon cell lines [9,26,27] and again that this occurs via a p53-independent route [26]. We have hypothesized that Bak upregulation is essential for butyrate-induced apoptosis [28]. Both p21 and Bak are up-regulated by reduction in Sp1 binding [9,29,30] and we now report here that acetylation of Sp1 following treatment of cells with the HDACi butyrate reduced Sp1 binding to p21 and bak promoter sequences. These data imply a common mechanism for cell cycle arrest and apoptosis following butyrate. Recent reviews of the mechanism of action of HDACi in general have been inconclusive, although other work has speculated that the cancer therapeutic actions of HDACi may also be a result of suppression of angiogenesis [31].

We hypothesized that up-regulation of p21 and Bak in response to the HDACi butyrate may have a common mechanism related to the biological regulation of their common transcriptional activator Sp1. Acetylation of Sp1 is a candidate mechanism for this regulation. Owing to the conflicted literature and potential physiological and pharmacological importance of this target, we investigated the effect of Sp1 acetylation on function in colon epithelial cells.

## Results

### Sp1 is acetylated in colon cells and its acetylation increases following treatment with the HDACi, sodium butyrate

Several previous reports indicate Sp1 acetylation is a determinant of DNA binding activity. In order to assess whether Sp1 is acetylated in the HCT116 colon cell line, the protein was immunoprecipitated (IP) using an anti-Sp1 antibody and the precipitate analysed by western blot and immunoprobing with anti-Sp1 and anti-acetyl lysine antibodies (see Additional File 1, Fig 1). This approach showed the predicted enrichment of Sp1 in the IP fraction. When the IP was immunoprobed with an anti-acetyl antibody, a cross-reaction occurred in the IP fraction at the same molecular weight as Sp1, suggestive of acetylation of this protein.

**Figure 1:**
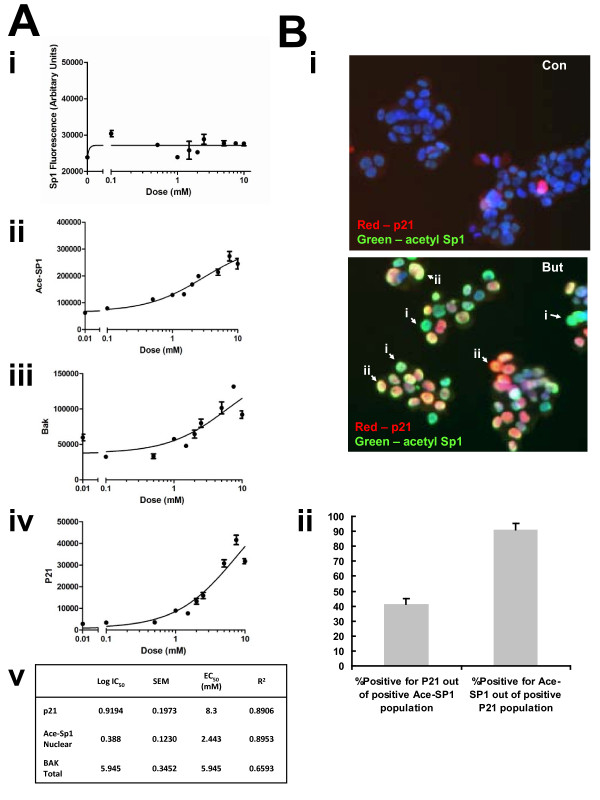
**Sp1 acetylation increases in a co-linear manner with bak and p21 expression following sodium butyrate treatment**. HCT116 cells were treated with increasing concentrations of butyrate (0-20 mM) for 24 hr, fixed and fluorescence immunostained either for Sp1 and Bak or Ace-Sp1 and p21. Cellular fluorescence was quantified by High-Content Analysis. Panel A shows protein expression levels of Sp1 (Panel Ai); acetyl-Sp1 (Panel Aii); Bak, (Panel Aiii) and p21 (Panel Aiv). Data are from a single pass experiment with three replicates, with 50 fields per replicate scored. The EC_50 _value for each event in response to butyrate is shown in Panel Av. Panel B shows representative images of HCT116 cells following 24 h of 0 or 10 mM sodium butyrate treatment and fluorescence immunostaining for acetyl-Sp1 and p21. Panel Bi shows representative fields from the control (untreated) cells (upper panel) and the treated culture (lower panel). Staining patterns in the treated cells broadly fell into two main types, cell positive for acetyl-Sp1 alone (examples marked by arrows i) and cells positive staining for both acetyl Sp1 and p21 (marked by arrows ii). These subpopulations were distinguished by plotting acetyl-Sp1 vs. p21 fluorescence (see supplementary online data Fig 2 for gating strategy). Panel Bii shows quantitation of data from three independent experiments, showing numbers of cells in gated fractions that were dual stained, as indicated.

In order to analyse Sp1 acetylation directly we generated an antibody to the published acetyl-Sp1 epitope (K703). The antibody was double-affinity purified (counter-purified against the non-acetyl epitope, then purified against the K703 epitope). The antibody (K703-Sp1), cross-reacted with a single band of the same molecular weight as Sp1 in whole cell lysates, did not cross-react in lines not expressing Sp1 and also detected immunoprecipitated Sp1 (see Additional File 1, Fig1).

The novel anti-acetyl-Sp1 antibody was used in a high-content analysis (HCA) approach to assess the effect of increasing concentrations of the HDACi butyrate upon Sp1 acetylation and expression of known Sp1 targets p21 and BAK. Relative Sp1, acetyl-Sp1, p21 and BAK expression levels were obtained using high-content analysis as described in the methods section. Sp1 expression levels were essentially constant (Fig 1Ai) following butyrate treatment, however acetyl-Sp1 levels increased in a concentration-dependent manner in response to butyrate treatment (Fig 1Aii). Expression of both Bak (Fig 1Aiii) and p21 (Fig 1Aiv) also demonstrated a concentration-responsive increase following butyrate treatment, and in line with Sp1 acetylation. The EC_50 _for Sp1 acetylation, Bak and p21 up-regulation are estimated in the table in Fig 1Av. The EC_50 _for all events are similar, particularly so for Sp1 acetylation and Bak up-regulation. The co-linearity of up-regulation suggests that both Bak and p21 up-regulation may be a consequence of Sp1 acetylation.

When images of cells dual-stained for acetyl-Sp1 and p21 were examined from cultures before and after butyrate treatment there was marked difference in level of acetyl-Sp1 cross-reaction and in expression and localisation of p21. In untreated cells there was little or no acetyl-Sp1 staining and most p21 cross-reactivity was cytosolic (Fig 1Bi). Following treatment with 10 mM butyrate for 24 h, there was a clear increase in acetyl-Sp1 staining, which was nuclear, and an increase in nuclear p21 staining. The merged image of p21 (red) and acetyl-Sp1 (green) staining revealed that most nuclei either appeared green (arrows marked i) or yellow-orange (arrows marked ii) with few or no nuclei staining red, indicating p21 colocalises with acetyl-Sp1 in the nucleus. In order to quantitate this observation, we plotted the percentage of cells positive for p21, which also stained for acetyl-Sp1 and vice versa (Fig 1Bii; the gating strategy is shown in the Additional File 1). This plot revealed that the majority (>91%) of p21-positive nuclei were also acetyl-Sp1 positive. In contrast, only 40% of acetyl-Sp1 positive cells were p21-positive, demonstrating that acetylation of Sp1 occurred without increased p21 expression. Taken together, we infer that following butyrate treatment, Sp1 acetylation precedes p21 up-regulation.

### Acetylation of Sp1 eliminates *in vitro *binding of promoter sites to two Sp1-regulated genes

**Figure 2:**
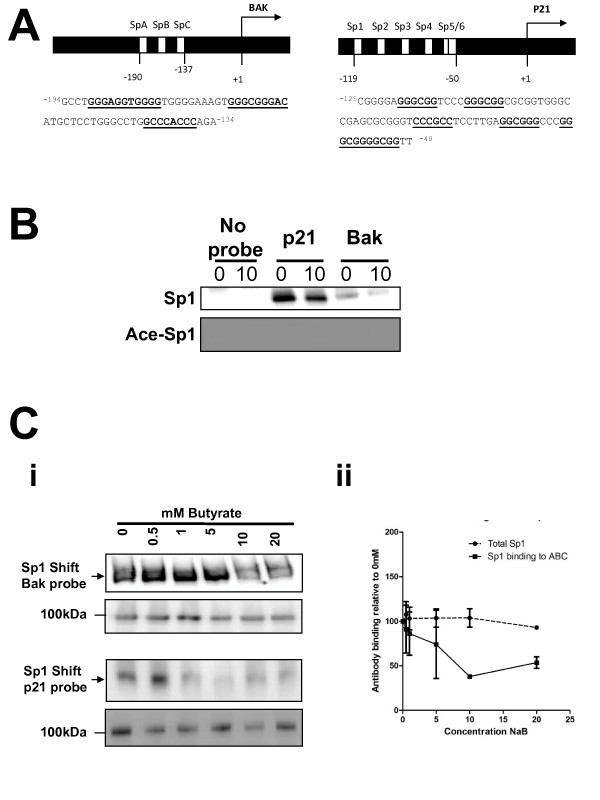
**Acetylated Sp1 does not bind at the bak or p21 promoters**. Panel A shows the organisation and sequence of the bak (Ai) and p21 (Aii) probes, with hypothetical and proven Sp1/3 binding sites underlined. Sequence numbers refer to distance from the transcriptional start site of each gene. Panel B shows that binding of Sp1 to both target sequences is decreased following butyrate treatment. Panel B: Western of mobility shift assay (WeMSA) analysis of binding to the Bak and p21 probes shows that Sp1 binding decreases following treatment with 10 mM sodium butyrate for 24 h compared to an untreated control (upper panel). Binding of acetyl-Sp1 could not be detected by WeMSA (lower panel). These data are representative of three independent repeats. Panel C: The binding of Sp1 to the bak (panel Ci) and p21 (panel Cii) promoter sequences was determined following treatment of HCT116 cells with a range of butyrate concentrations (0-20 mM). The upper panels show WeMSA gels immunoprobed for Sp1. As a loading control, the same extracts were also separated by SDS page, and immunoprobed with the same antibody (lower panels). Data shown in Ci and Cii are representative of at least two independent repeat experiments. Panel Ciii shows mean (+/- SD) of response at the Bak promoter. Whilst the levels of Sp1 are broadly constant, levels of Sp1 binding for both probes are reduced following treatment with butyrate.

The location of K703 in the Sp1 DNA-binding domain led us to ask whether acetyl-Sp1 retains its DNA-binding activity. To test the DNA-binding ability of acetyl-Sp1 we used sequences from the Bak and p21 promoters, which are known to bind Sp1 [9,32]. These sequences contain several potential and confirmed Sp1/3 binding sites (Fig 2A). Western blots of mobility shift gels (WeMSAs) were undertaken before and after butyrate treatment with both bak and p21 probes. When mobility shift gels were immunoprobed for Sp1, a characteristic cross-reaction was observed (Fig 2B), which decreased with both probes after butyrate treatment. When identical gels were probed with anti-acetyl-Sp1 antibody no cross-reactions were ever observed, despite repeated efforts. The WeMSA method works in our hands (as we can get cross-reaction with Sp1, or Sp3) and the acetyl-Sp1 antibody appears to work in some applications (as conventional SDS-PAGE immunoblotting works with the antibody), and so we interpret these data as showing that the intracellular pool of acetyl-Sp1 has little or no binding affinity for the *Bak *or *p21 *promoters, although other Sp1 species may retain binding affinity.

We undertook a concentration-response study to establish the range of butyrate concentrations which would cause a reduction in Sp1 binding and to establish whether any underlying alteration in Sp1 levels may be associated with the observed decrease in binding. Nuclear extracts prepared from 0-20 mM butyrate treated cells were assayed for bak and p21 promoter binding activity using the same approach. Total levels of Sp1 expression were also measured by western blotting the extracts. Data are shown in Fig 2C. Increasing butyrate concentration caused a progressive decrease in binding of Sp1 to both the *bak *(Fig 2Ci - upper panel) and *p21 *(Fig 2Cii - upper panel) target sequences. The reduction in binding could not be explained by significant reduction in Sp1 basal levels, which were broadly unaltered by butyrate (Fig 2Ci and 2Cii lower panels). These data are representative of two independent repeats. We noted that the relative concentration required to reduce the binding of Sp1 to the promoter was lower for p21 that for BAK. This may be a consequence of differing affinity of Sp1 for these sequences, or a sub-optimal choice of target sequence for the assay. When trying to undertake loading validation with a series of standard loading controls we noted many of the proteins used to standardise loading of nuclear extract were themselves altered by butyrate, although underlying protein levels were constant as measured by coomassie-stained gels (these data are shown in Additional File 1, Fig 3).

**Figure 3:**
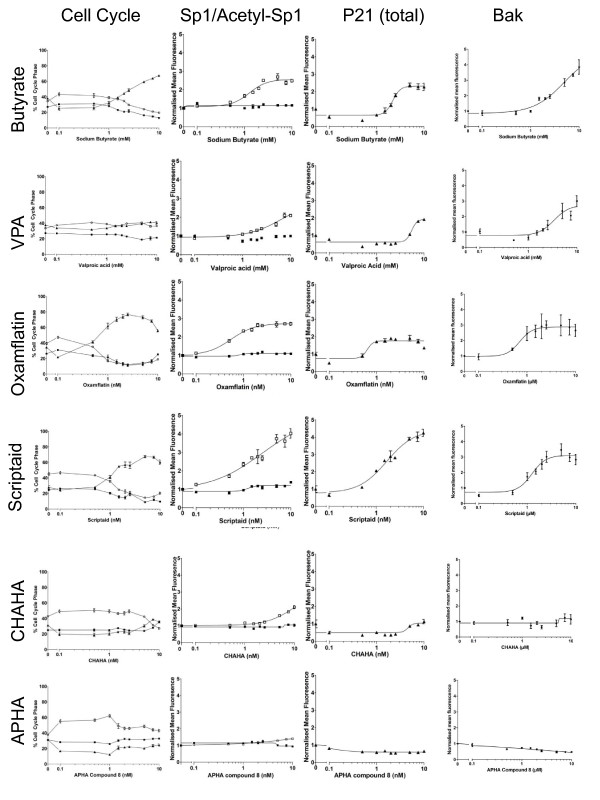
**Effect of HDACi on cell cycle, p21 expression, bak expression and Sp1 expression and acetylation**. The extent of the concomitant response of Sp1 acetylation, cell cycle arrest and p21 up-regulation was determined using a high-content biology approach. HCT116 cells were treated with concentration ranges of 0-20 mM sodium butyrate, 0-20 mM valproic acid (VPA), 0-20 μM Oxamflatin, 0-20 μM Scriptaid, 0-20 μM APHA compound 8, 0-20 μM CHAHA. In all cases treatments were carried outfor 24 h. Cells were stained using immunocytochemistry for DNA content (Hoescht), p21, bak, Sp1 and acetyl-Sp1 as described in the methods section. Cells were analysed, on the basis of DNA content, for cell cycle phase and divided into G1 (filled circles), S (filled squares) or G2/M (filled triangles). Levels of protein were calculated from mean total fluorescence and are expressed in terms of fluorophore fluorescence relative to that observed in untreated cells. Sp1 (filled squares) and acetyl-Sp1 (open squares) are shown on the same graph.

### Several HDACi induce cell cycle arrest in G2/M, at concetrations associated with Sp1 acetylation and p21 and Bak upregulation

In order to address whether the response to butyrate was representative of the effects of HDACi, we examined the cellular response to other HDAC inhibitors. The HDACi in this study are listed in the Additional File 1, Table 1, and include a variety of HDACi structure classes and inhibition profiles. The five HDACi selected for this study were butyrate, valproic acid (VPA), oxamflatin, scriptaid, 3-(1-Methyl-4-phenylacetyl-1H-2-pyrrolyl)-N-hydroxy-2-propenamide (APHA compound 8) and the SAHA analogue, (2*E*,4*E*)-6-(4-Chlorophenylsulfanyl)-2,4-hexadienoic acid hydroxyamide (CHAHA). These HDACi were used to treat HCT116 cells across a range of concentrations chosen on the basis of published data and spanning a 2-log scale for each compound. Cells were treated for 24 h, then fixed and stained as described in the methods section before analysis by HCA. Multiple cellular outcomes (cell cycle, p21 expression, Bak expression, PARP cleavage, Sp1 expression and Sp1 acetylation) were measured in a single pass of the experiment, which included 3 internal replicates.

Our previous work on butyrate has indicated an accumulation of cells in G2/M following treatment with concentrations above 0.5 mM [34] although there are also publications to suggest that butyrate triggers arrest in G1 [31,33]. In this study we found clear evidence of G2/M phase arrest with butyrate in the 1-2 mM range which increased with drug concentration up to 10 mM without obvious saturation (Fig 3). In marked contrast the branched chain fatty acid (BCFA) valproic acid (VPA) seemed to have no effect in this concentration range with no marked alteration in cell cycle profile even up to treatment at 10 mM. Four different hydroxamic acid derivatives were used in this study: oxamflatin, scriptaid, CHAHA and APHA compound 8. Even within a group of HDACi with common conserved molecular origin, there were distinct differences in the effects upon cell cycle: oxamflatin and scriptaid both induced a G2/M arrest in cells, whereas CHAHA and APHA compound 8 induced a less pronounced G1 arrest. We noted that there was a peak and the strength of effect was reduced at high doses for oxamflatin and scriptaid. The G1 arrest triggered by APHA compound 8 was only clear at low doses and at higher doses no effect was noted.

Concomitant with analysis of effects on cell cycle and identification of distinct responses from subsets of HDACi, the response of p21 to multiple HDACi was tested. Cells were treated over the established concentration range and p21 levels determined by HCA. Several of the HDACi induced p21 expression in a concentration responsive manner, with maximum effects observed at the highest doses: butyrate induced a 2.27 (+/- 0.20 SEM) average increase in p21 relative to untreated cells; valproic acid (VPA) also induced a near 2 fold increase (mean 1.91+/-0.08 SEM); the hydroxamic acid, scriptaid produced a much larger average relative increase in p21 of 4.25+/-0.21 SEM at the maximum dose used; oxamflatin, also increased p21 in a concentration-responsive manner, however the effect peaked at 5 nM (mean 1.91 fold increase +/- 0.16 SEM) and was reduced at higher doses, indicating potential toxicity. Treatment of HCT116 cells with APHA compound 8 had no effect on p21 expression at the concentrations used. Low concentrations of CHAHA treatment produced a slight decrease in p21 protein levels relative to untreated cells, which corrected to baseline at higher doses.

The response of Bak to HDACi was also examined. As was the case for p21 and cell cycle, cells were treated over the established concentration range and Bak protein levels determined by HCA. The HDACi produced similar effects on Bak expression as those seen for p21. VPA, Scriptaid and oxamflatin produced increases of approximately 3 fold in Bak expression. Butyrate induced a marginally larger increase in Bak protein levels of 3.5 fold, compared to untreated cells. APHA compound 8, consistent with the results for p21, produced no significant change in expression. CHAHA treatment produced a negligible decrease in expression, similar to that observed for p21. Transcription of both p21 and Bak is known to be regulated by Sp1. Therefore we examined the levels of total Sp1 and acetylated Sp1 using the acetylated K703-Sp1 antibody. Levels of Sp1 protein remained constant following treatment with all of the HDAC inhibitors tested (see Fig 3). In contrast Sp1 acetylation increased in response to several HDACi, notably scriptaid (maximum concentration produced a 4.02 fold mean increase +/-0.25 SEM), butyrate and oxamflatin (maximum concentrations produced 2.49 fold mean increase +/-0.08 SEM and 2.71 +/-0.09 SEM respectively) and CHAHA and VPA (maximum concentrations resulted in 2.10 fold mean increase +/-0.09 SEM and 2.10 +/-0.04 SEM). There was little detectable response of Sp1 acetylation to APHA except at the highest concentration which increased Sp1 acetylation by a modest mean 1.4 fold (+/- 0.04 SEM).

**Figure 4:**
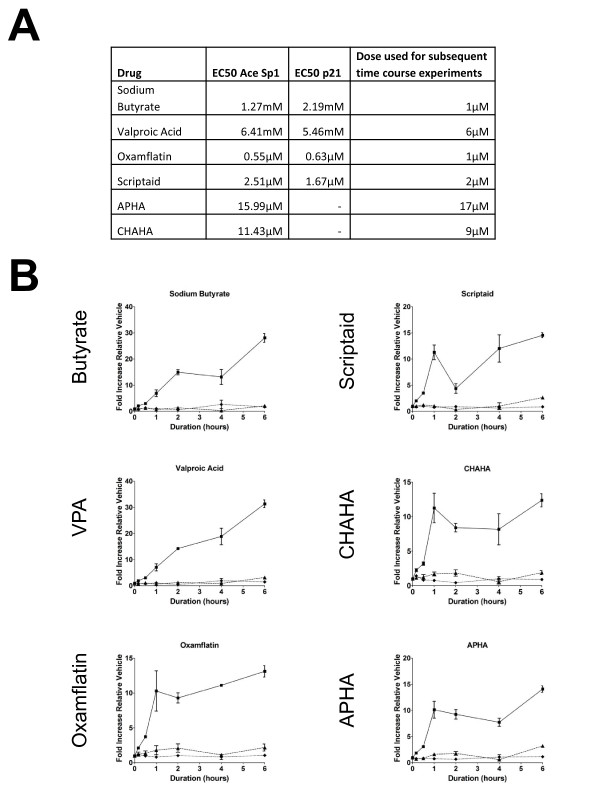
**Acetylation of Sp1 precedes p21 up-regulation in response to all HDACi**. Panel A shows EC_50 _values calculated from the concentration-response data shown in Fig 2, for each of Sp1 acetylation and p21 upregulation. There was generally close agreement between these values. The third column indicates the concentration used in subsequent timecourse experiments. Panel B shows timecourse experiments. HCT116 cells were treated with concentrations of HDACi as indicated in panel A, for times from 0 to 6 h. Timepoints less that 1 h are 10 min and 30 min. Cells were fixed and stained for acetyl-Sp1 and for p21 as before. Fluorescences were quantitated using high content approaches. Graphs are for acetyl-Sp1 (filled squares) p21 (filled triangles) and total Sp1 (filled diamonds). The results show the means of three repeat experiments with internal triplicates.

To assess whether the HDACi were inducing apoptosis at this timepoint, PARP cleavage was scored in response to all concentrations of each drug used. Whilst most of the HDACi induced apoptotis at levels of 2-5% and little over background, oxamflatin and scriptaid induced PARP cleavage in 10-15% of cells, suggesting a potentially faster mechanism of action that we and others have previously shown for butyrate [9,34]. Data are shown in the Additional data file, Fig 4.

### Acetylation of Sp1 precedes p21 upregulation in response to all HDACi

There was a notable concomitance between the observed effects of the HDACi group on Sp1 acetylation, p21 expression and G2/M phase cell cycle arrest. The EC_50 _for each event and for each compound was calculated and is shown in Fig 3A. There is also similarity between the EC_50_s for Sp1 acetylation, p21 up-regulation and G2 arrest.

Following the concentration-response experiments, concentrations of the HDACi approximating to the EC50s for cell cycle arrest, Sp1 acetylation and p21 upregulation were chosen, as indicated in Fig 4A, for use in a timecourse study. As p21 plays an important role in regulation of the cell cycle we anticipated that its expression would vary as the cells approached confluency. Therefore all experiments were carried out on subconfluent cells. When carrying out a time course we also chose to look at 0-6 hours of treatment to identify early changes preceding, rather than consequential to, cell cycle impairment. The time course (see Fig 4B) demonstrated that all HDACi induced an increase in acetylated Sp1 when compared to a time matched control. There was no increase in total Sp1 cross-reactivity, confirming it was a change in acetylation being observed rather than increased expression. This increase in acetylation of Sp1 was a very rapid event with a clear increase observable in as little as 10 minutes of treatment. The up-regulation of p21 was examined across the same period. There was no increase in p21 expression in response to any of the HDACi with 0-6 hours of HDACi treatment. These data agree with the findings presented in Fig 1 that Sp1 acetylation may precede p21 up-regulation. The data suggest that, in contrast to the 24 h time-point used in the concentration-response experiment (Fig3), the HDACi tested all induce Sp1 acetylation but that this induction is transient for some compounds (notably CHAHA and APHA) and, given the stark differences in cell cycle events, the differential effect results in downstream activation of distinct pathways.

### Mimicking Sp1 acetylation using siRNA knockdown targets the p53/p21 pathway

**Figure 5:**
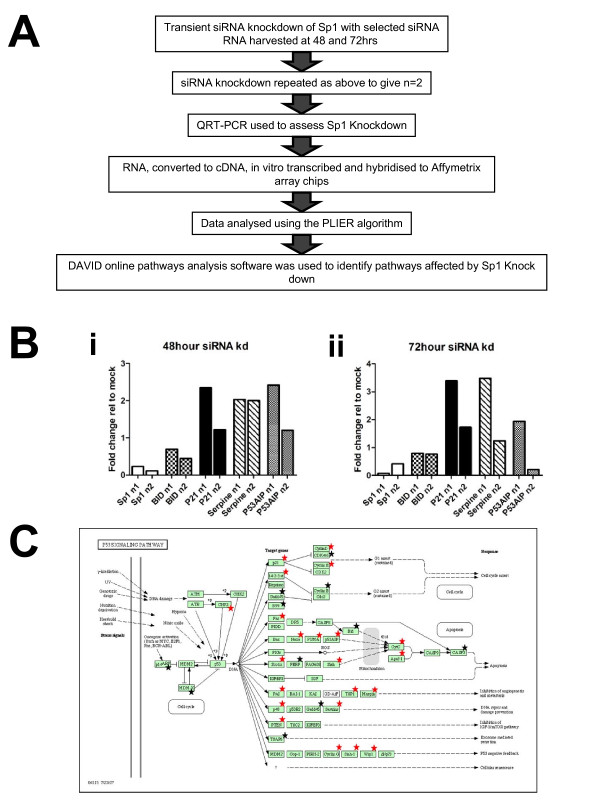
**Knock-down of Sp1 reveals multiple targets in the p53 signalling pathway are altered**. Sp1 knockdown was achieved using Ambion siRNA. RNA was extracted 48 and 72 hours post transfection, labelled and hybridised to human U133 plus 2.0 arrays. Analysis of microarray data was carried out using GCOS, Array assist and the DAVID online analysis tool. Statistical significance was determined using an unpaired two-tailed t test, summarised in the workflow shown in Fig 5A. Knockdown was confirmed by QPCR using TaqMan^® ^gene expression assays (Fig 5B). Due to variability in knockdown produced by transient transfections, the biological replicates are shown as individual bars. The gene list identified using PLIER was sorted for statistically significant (p > 0.05) gene expression changes of greater than 1.2 fold. Subsequent analysis of this list using DAVID identified a number of changes occurring in the p53/P21 regulatory pathway (panel C; adapted from DAVID [[39,40], and http://www.genome.jp/kegg/). Stars denote genes which were significantly upregulated (red) or down-regulated (black). Validation of a subset of genes was carried out using Applied Biosystems TaqMan^® ^gene expression assays, according to manufacturer's instructions. QPCR expression data for Bid, p21, serpine, P53AIP and Sp1 are shown in Fig 5, panel B for HCT116 cells following Sp1 knockdown harvested 48 (panel Di) or 72 (panel Dii) hours post-transfection. The biological replicates are shown as individual bars; designated n1 and n2.

Our experiments indicated that acetylation of Sp1 at K703 altered the binding affinity of Sp1 by abolishing binding activity to the Bak and p21 promoters. We next sought to investigate what effects acetylation of Sp1 might have on the wider regulation of genes whose expression was regulated by Sp1. Initially we intended to use siRNA mediated knockdown of HDACs to identify the effector of Sp1 acetylation and to increase acetylation of Sp1. However our work indicated that siRNA knockdown of HDACs induced compensatory mechanisms upregulating expression of other HDACs (data not shown). Therefore to identify further gene targets of Sp1 affected by acetylation we used siRNA knockdown of Sp1 to mimic the abolished Sp1 binding observed following acetylation. The workflow for this study is shown in Figure 5A.

Three predesigned Sp1 siRNAs from Ambion were tested for efficiency of knockdown, off- target effects and alterations in cell growth. The most effective oligonucleotide, which did not noticeably affect cell growth, was chosen for subsequent experiments. This siRNA was used to transfect HCT116 cells. Success of the transient Sp1 knock-down was verified by q-RT-PCR for Sp1 (Figure 5B). Two biological replicates of the experiment were used for a microarray analysis. The average percentage of genes described as present on the GeneChips for the 48 h and 72 hour mock transfected samples were 40.05% and 41% respectively. Sp1 knockdown samples produced a similar level of present calls with an average of 40.8% and 39.3%, at 48 and 72 h post transfection. Expression analysis was carried out using the PLIER algorithm within the ArrayAssist programme (Affymetrix). Probes whose signal intensities were below the average background level were disregarded. A gene list was compiled of genes with a p value of ≤0.05 and a fold change of ≥1.2, relative to the mock control for each time point (see Tables 1 and 2). These differentially expressed genes were analysed using the functional annotation tool of the DAVID Bioinformatics Resource. This analysis identified a number of pathways which contained a significant number of differentially expressed genes including: p53 signalling, cancer, apoptosis and cell cycle pathways. The p53 signalling pathway was the only pathway which was identified as being altered in both the 48 h and 72 h datasets. Therefore for the purposes of this study we focused our subsequent analysis on the p53 signalling pathway, which included p21, consistent with our earlier findings. The approximately 2 fold upregulation in p21 expression observed by the microarray analysis was confirmed by QPCR (Fig 5D). This QPCR analysis highlighted the variation in expression changes between biological replicates; this is likely due to the heterogenous nature of transient siRNA knockdown cultures. However both biological replicates showed increased p21 expression relative to the time matched, mock-transfected controls for each timepoint. To validate further the microarray results, the expression of three other genes from the p53/p21 pathway, Bid, Serpine and P53AIP, were also checked by QPCR (Fig 5D). In concurrence with the microarray data, both biological replicates showed downregulation of Bid following Sp1 knockdown at both 48 (69.7% and 44.4% of mock expression) and 72 hours (79.4%; 76.5% of mock expression) post transfection (Sp1 knockdown increased Serpine mRNA levels at 48 h post tranfection to 2.03 fold (replicate 1) and 2.00 fold (replicate 2) of those observed in mock transfected samples. Serpine mRNA expression levels were also increased at 72 hours post Sp1 knockdown, however the biological replicates showed considerable variation: replicate 1, 3.48% increase relative to mock; replicate 2, 1.24% increase relative to mock. The variation in fold changes for Serpine, observed between the biological replicates, again reflects the heterogenicity of transient siRNA knockdown cultures.

P53AIP showed variable levels of up regulation 48 hours post knockdown (1.2-2.4 fold up). At 72 hours post Sp1 knockdown P53AIP mRNA expression was increased by 1.94% relative to mock in biological replicate 1 (n1) but decreased by 79.2% in the second replicate (n2). These contradicting data may reflect greater restoration of function in one replicate as Sp1 expression is higher in replicate 2, indicating that the Sp1 siRNA knockdown is in decline.

## Discussion

Numerous *in vitro *studies have shown that butyrate, at physiological concentrations, triggers cell cycle arrest and apoptosis, observations which underpinned the hypothesis proposed by the EPIC team - that butyrate is a principal chemopreventive effector of fibre consumption. Butyrate is an HDACi at physiological concentrations and although there is considerable interest in the development and application of HDACi in cancer therapy and prevention, the underlying mechanisms of action remain unclear. Work addressing the molecular pharmacology of cell cycle arrest showed a p53 independent activation of p21 expression was a central event in cell cycle arrest [30,35-37]. In studies addressing the molecular mechanisms by which butyrate induces apoptosis, we noted that this appeared to be independent of cell damage and resultant signalling. We proposed a model whereby Bak upregulation by butyrate is a key contributory mechanism in the cancer-preventive properties of fibre [9]. More recent *in vivo *studies have confirmed a central role for Bak in colorectal carcinogenesis in mice [38]. The up-regulation of both Bak and p21 by butyrate appears to be due, at least in part, to inhibition of promoter binding by Sp1, allowing access to the promoter region by Sp3 to drive transcription. How might such a change in binding be effected? We showed that binding of Sp1 to its target sequence site is diminished following butyrate treatment, in a concentration-responsive manner. A new antibody to acetyl-Sp1 shows that acetylation of Sp1 increases in a concentration-dependent manner in response to butyrate exposure.

An HCA approach was used to determine the dose-response curves for Sp1 acetylation and Bak up-regulation. The curves are very similar, resulting in similar EC_50 _values. In contrast, the p21 curve was shifted to the right and gave a higher EC_50 _value, although further work is required to determine whether real EC_50 _differences occur between Sp1, Bak and p21. However, the difference in p21 EC_50 _could be attributable to a composite effect of p21 transcriptional up-regulation, and nuclear relocalisation. The p21 promoter is also more complex relative to the bak promoter, therefore EC_50 _differences may reflect differing Sp1/Sp3 binding potentials at these binding sites. The gating analysis suggests that Sp1 acetylation precedes p21 up-regulation. We therefore hypothesize that p21-upregulation is mediated, at least in part, through the decreased binding of the p21 promoter by Sp1, perhaps allowing access to a weaker affinity stronger transactivator. We and others have previously hypothesized that Sp3 may fulfil such a function.

We assessed the effect of multiple members of the HDACi family on cell cycle progression and on expression of p21. Cell cycle arrest associated with p21 is more frequently associated with G1 arrest. Our data indicate that a G2/M arrest is consistently observed with several of the HDACi used. The pattern of cell cycle arrest did not correlate with compound class, with subsets of the SCFA and hydroxamic acids triggering G1 arrest. Whilst our findings do not imply causality, there is consistency between p21 upregulation and G2/M arrest as indicated by similar EC_50_. In contrast compounds which triggered primarily G1 arrest did not induce p21 expression. This may imply value in investigation of further roles for p21 at other phases of cell cycle. The degree to which each HDACi may sustain alterations in Sp1 acetylation (differences were more pronounced at 24 h than 6 h) could be a contributory factor to differences observed. For example APHA and CHAHA both triggered an observable alteration in Sp1 acetylation at 6 h, but in contrast to other hydroxamic acids the effect had passed by 24 h. It may be that the persistence of Sp1 acetylation determines the pattern of cell cycle arrest.

Our microarray analysis following Sp1 knockdown revealed that reducing Sp1 promoter occupancy by siRNA knockdown altered the regulation of a number of genes involved the p53 signalling pathway. These data indicate that reduced promoter occupancy by Sp1, similar to that observed following acetylation of Sp1, can influence cell cycle/death decisions. We noted in our analysis of the array data that Bak was not altered sufficiently to reach the threshold for inclusion in the analysis. Our previous data [9] showed that at the transcriptional level, changes in Bak mRNA were modest, but consistent across several assays and we hypothesized that this was sufficient to unbalance the cell and drive apoptosis. Other genes, for example Bid, were identified as larger fold changes in this study and may synergise with alterations in Bak to yield an apoptosis-susceptible cell. Furthermore, these data demonstrate that p53 controlled pathways can be regulated by alteration of Sp1 promoter occupancy, indicating that a complex interaction occurs between these two transcription factors.

The colon epithelial cell exists bathed in high levels of butyrate. Cell turnover rates in the colon are high with movement from the stem cell to apoptosis from the flat musosa in 3-4 days. During this period the cell will proliferate, arrest, differentiate and die, relying on butyrate to drive the sequence of these events through a highly coordinated set of transcriptional responses. Low levels of butyrate, as may be the case in cancer-prone low-fibre consumers, would result in lower levels of Sp1 acetylation, resulting in less cell death and more proliferating cells in the colon as a consequence of reduced Bak and p21 expression. A second pro-carcinogenic pathway could be associated with the low-butyrate setting: the lower expression of pro-apoptotic protein, would result in a cell less likely to die in response to a fixed amount of cytotoxic damage. These pathways (impaired physiological cell turnover and reduced ability to respond to damage) could contribute to increased cancer risk. One limitation of this study is that it is undertaken *in vitro *with a cancer-derived cell line. Our ongoing studies are testing the hypothesis raised - that Sp1-HDAC interaction may be central to the cancer preventive actions of butyrate through engagement of specific target genes - in cross sectional studies involving human volunteers. Our work thus far highlights the key role acetylation plays in the regulation of colonocyte cell cycle. Furthermore there is a need for specific HDAC inhibitors for the treatment of cancers arising in this cell population.

## Conclusions

• Acetylation of Sp1 reduces affinity for the Bak and p21 promoters, leading to upregulation.

• Sp1 is acetylated in response to multiple HDACi.

• Acetylation of Sp1 may represent a common mechanism for induction of cell death and cell cycle arrest pathways.

## Methods

### Cell culture

HCT116 cells were used throughout. Cells were grown in 1 g/L glucose DMEM (Gibco), supplemented with 10%v/v FCS (BioSera, E. Sussex, UK), 0.1 mg/ml streptomycin and 100 U/ml penicillin (Gibco). For treatment with butyrate, cells were grown to 40-50% confluency. Growth medium was discarded and replaced with 1 g/L glucose DMEM (Gibco, Paisley, Scotland), supplemented with 10%v/v FCS, 0.1 mg/ml streptomycin, 100 U/ml penicillin (Gibco) and 0-20 mM sodium butyrate (Calbiochem, Nottingham, UK).

### High-Content Analysis

HCT116 cells were seeded in 96 well plates at 8 × 10^3 ^cells per well. 24 hours after seeding the medium was replaced with growth medium supplemented with 0-10 mM sodium butyrate. Cells were treated for 24 hours before being fixed in 3.7% formalin and stained for Bak and Sp1 or acetyl-Sp1 and p21. All antibodies were diluted in 500 μg/ml digitonin/PBS solution according to Imagen Biotech proprietary HCA protocols. Antibodies used were: Sp1 (Cat# 07-645, Millipore), p21 (Cellomics p21 Kit, Thermo Fischer), Bak (Cat# 556396, BD Biosciences) and a custom antibody to acetylated Sp1. Cross reactions were visualised using fluorophore conjugated seconday antibodies: donkey anti-mouse (Alexa fluorophore 488 - green) and donkey anti-rabbit (Alexa fluorophore 555 - red). DNA was stained with Hoechst 33342 at 2.5 μg.mL^-1^. Plates were analysed on a Cellomics Arrayscan. The Arrayscan Compartmental Analysis algorithm was used to generate, a mask to measure either cytoplasmic or nuclear staining for each fluorescent signal.

### Protein Methods

#### Protein extraction and quantitation

Following treatment, nuclear extracts for DNA binding assay were prepared using Active Motif nuclear extract kit (cat#40010, Active Motif, Rixensart, Belgium) as per manufacturer's instructions. Cells for whole cell lysate extraction were washed twice in PBS, and lysed in lysis buffer (50 mM Tris, pH 7.4, 1 mM EDTA, 150 mM NaCl, 1 mM sodium orthovanadate, 0.5% NP-40, protease inhibitors (0.1 mM phenylmethylsulfonyl fluoride, Sigma protease inhibitor cocktail), and 100 mM sodium butyrate to inhibit HDAC activity. All reagents were from Sigma (Poole, UK) except for butyrate, which was as above.

Protein concentrations were quantified using the BioRad protein assay (cat#500-0006, BioRad, Hertfordshire, UK) as per manufacturer's instructions.

#### Western blotting

Proteins were separated on SDS-PAGE gels and transferred to PVDF for immunoprobing. After blocking nonspecific binding sites overnight with 5% nonfat milk in TBST (Sigma), the membrane was incubated for 1 h at room temperature with primary antibody solutions in block. The membranes were subject to 3 × 10 min TBST washes following each antibody incubation. Antibodies used include: HRP conjugated mouse anti-Actin (ab20272, Abcam), rabbit anti-Sp1 (cat#07-645, Millipore), rabbit anti-Sp3 (D-20, Santa Cruz Biotechnology); rabbit anti-acetyl lysine (cat#ab3879, Chemicon); mouse anti-HDAC1 (cat#05-614, clone 2e10, Millipore); mouse anti-HDAC2 (cat#05-814, clone 3F3, Millpore); rabbit anti-HDAC3 (ab16047, Abcam). Cross-reactions were visualized using HRP-conjugated secondary antibodies (Dako, UK), Immobilon Western HRP substrate (Millipore, UK) and a Chemigenius BioImaging system (Syngene, Cambridge, UK). The antibody to acetylated K703 of Sp1 was commissioned from Eurogentec to the sequence previously established [3]. The antibody was double-affinity purified - both counter purified against the non-acetyl epitope and positively purified against the acetyl epitope.

### DNA-binding assays

Electromobility shift assays (EMSAs) were conducted using the lightshift EMSA kit from Pierce (Cat#20148, Pierce, Rockford IL USA). An adapted version of the EMSA protocol, a western of a mobility shift gel (WeMSA) was carried out as previously described [9]. Briefly: unlabelled oligonucleotides were incubated with nuclear extracts as per Lightshift EMSA kit instructions; complexes were separated by molecular weight using 5% TBE acrylamide mini-gels in 0.5 × TBE; gels were incubated in SDS buffer (25 mM Tris; 192 mM glycine; 0.2% (w/v) SDS) for 10 min prior to being transferred to PVDF at 100 V for 1 h in 0.5 × TBE; membranes were blocked in 5% milk TBST for 1 h prior to immunoprobing and ECL detection of HRP conjugated secondary antibodies. Oligonucleotides for binding assays were commissioned from Sigma Genosys. Oligonucleotides used for EMSA were 3' biotin-labelled.

### siRNA delivery

#### Sp1 knockdown

Our initial siRNA experiments identified problems with the commercial negative control and showed that mock transfected cells were a better control. Therefore for the microarray experiments cells were transfected with Sp1 (Cat No. AM16704, ID 143158) siRNA or mock transfected. Transfections were carried out at the time of plating (forward transfection) using Lipofectamine RNAi max (Invitrogen), according to the manufacturer's protocol for transfecting 24 well plates. 3 × 10^4 ^HCT116 cells and a final siRNA concentration of 10 nM were used. Twelve wells for each transfection condition were transfected to ensure enough RNA was available for both QPCR and microarray analysis, these were pooled prior to RNA extraction. Samples were collected 48 h and 72 h post transfection to examine the downstream effects of Sp1 knockdown. Trizol reagent (Invitrogen) was used to extract total RNA.

### Bioinformatic Approaches

#### RNA Quality checks

RNA quantity was determined using a NanoDrop 1000 spectrophotometer (Labtech International, East Sussex, UK). The 2100 bioanalyzer,RNA 6000 Nano LabChip (Agilent, Palo Alto, CA) was used to assay RNA integrity and samples were only taken forward if the quality was satisfactory as indicated by the absence of ribosomal RNA degradation.

#### Microarray analysis

Double stranded cDNA was synthesised and then i*n vitro *transcribed to produce biotin-labeled cRNA (GeneChip Expression 3_-Amplification reagents for *in vitro *transcription labeling; Affymetrix, Santa Clara, CA). The amplified cRNA wasthen analyzed for quality (Agilent 2100 Bioanalyzer, RNA 6000 NanoLabChip) and quantity (NanoDrop 1000 Spectrophotometer). 15 μg of cRNA was fragmented (Gene-Chip reagents; Affymetrix) and hybridized to Human Genome U133 Plus 2.0 GeneChip. Four chips (2 mock transfected; 2 Sp1 siRNA transfected) were hybridized for each time point according to Affymetrix protocols. After overnight hybridizationat 42°C, the GeneChips underwent stringency washes in a GeneChip Fluidics Station 400 (Affymetrix) and were scanned with a laser at high resolution (GeneChip Scanner 3000; Affymetrix). The results were analyzed initially using GeneChip operating software (GCOS), which automatically acquires and analyzes image data and computes an intensity value for each transcript. The data were subsequently processed using ArrayAssist (Iobion Informatics, La Jolla, CA) to statistically analyze changes in gene expression in the presence of the Sp1 knockdown at each time point.

Transcripts were defined as differentially expressed between mock and Sp1 siRNA transfected cells if there was a 1.2 fold or greater difference in the gene expression level, plus a *p *value of less than 0.05. The statistical test applied by the ArrayAssist program was an unpaired two-tailed *t *test.Differentially expressed probe sets were classified according to their molecular function, biological process, cellular compartment and chromosomal location using GeneOntology terms. To identify specific pathways affected by Sp1 knockdown, the DAVID bioinformatics database was also used http://david.abcc.ncifcrf.gov/home.jsp[39,40].

#### Quantitative PCR

Prior to reverse transcription amplification grade DNAse (Invitrogen) was used to eliminate any genomic DNA contamination. Reverse transcription was carried out using the superscript III reverse transcriptase (Invitrogen) and random hexamers (Promega) as per manufacturers' instructions. QPCR was used to confirm Sp1 knockdown prior to microarray hybridisation and to validate gene changes as identified by microarray analysis.

The ΔΔCt real time PCR method [41] was used to quantitate gene expression using Applied Biosystems Taqman gene expression assays and mastermix. The protocol used was carried out as per manufacturers instructions in 20 μl reactions.

## Competing interests

The authors declare that they have no competing interests.

## Authors' contributions

JSW undertook the majority of the experiments, contributed to experimental design and wrote part of the manuscript; HC undertook some of the experiments, and jointly conceived the study; CWY undertook some of the experiments; GJG completed preliminary analysis of HCA data; RSPB undertook analysis of HCA data; CDB jointly conceived the study, contributed to experimental design and interpretation; BMC conceived and directed the study, interpreted data and wrote the manuscript.

All authors read and approved the final version of the manuscript.

## Supplementary Material

Additional file 1 **Data includes diagrams of structures of HDACi used in this study, antibody validation work, gating strategy for HCA analysis, additional controls supporting Fig 4B, and graphs of levels of apoptosis triggered by HDACi used in this paper**. Click here for file
