# Supplementary material for: Sp1 acetylation is associated with loss of DNA binding at promoters associated with cell cycle arrest and cell death in a colon cell line
Source: Mol Cancer. 2010 Oct 15;9:275. doi: 10.1186/1476-4598-9-275 (PMC2972244; doi:10.1186/1476-4598-9-275)
Supplement: Additional file 1 — Data includes diagrams of structures of HDACi used in this study, antibody validation work, gating strategy for HCA analysis, additional controls supporting Fig 4B, and graphs of levels of apoptosis triggered by HDACi used in this paper. [file 1476-4598-9-275-S1.ppt]

## Slide 1
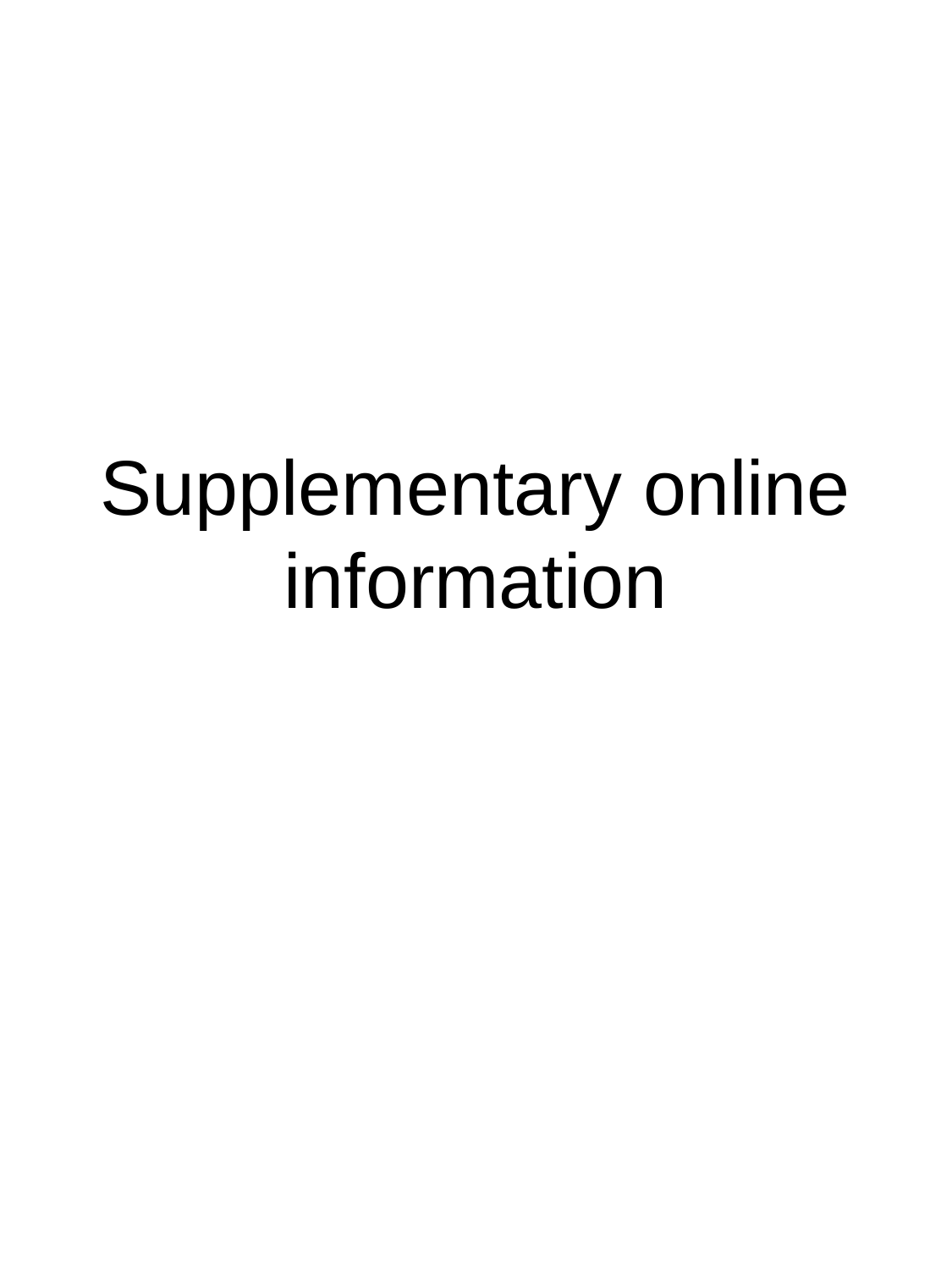

# Supplementary online information

## Slide 2
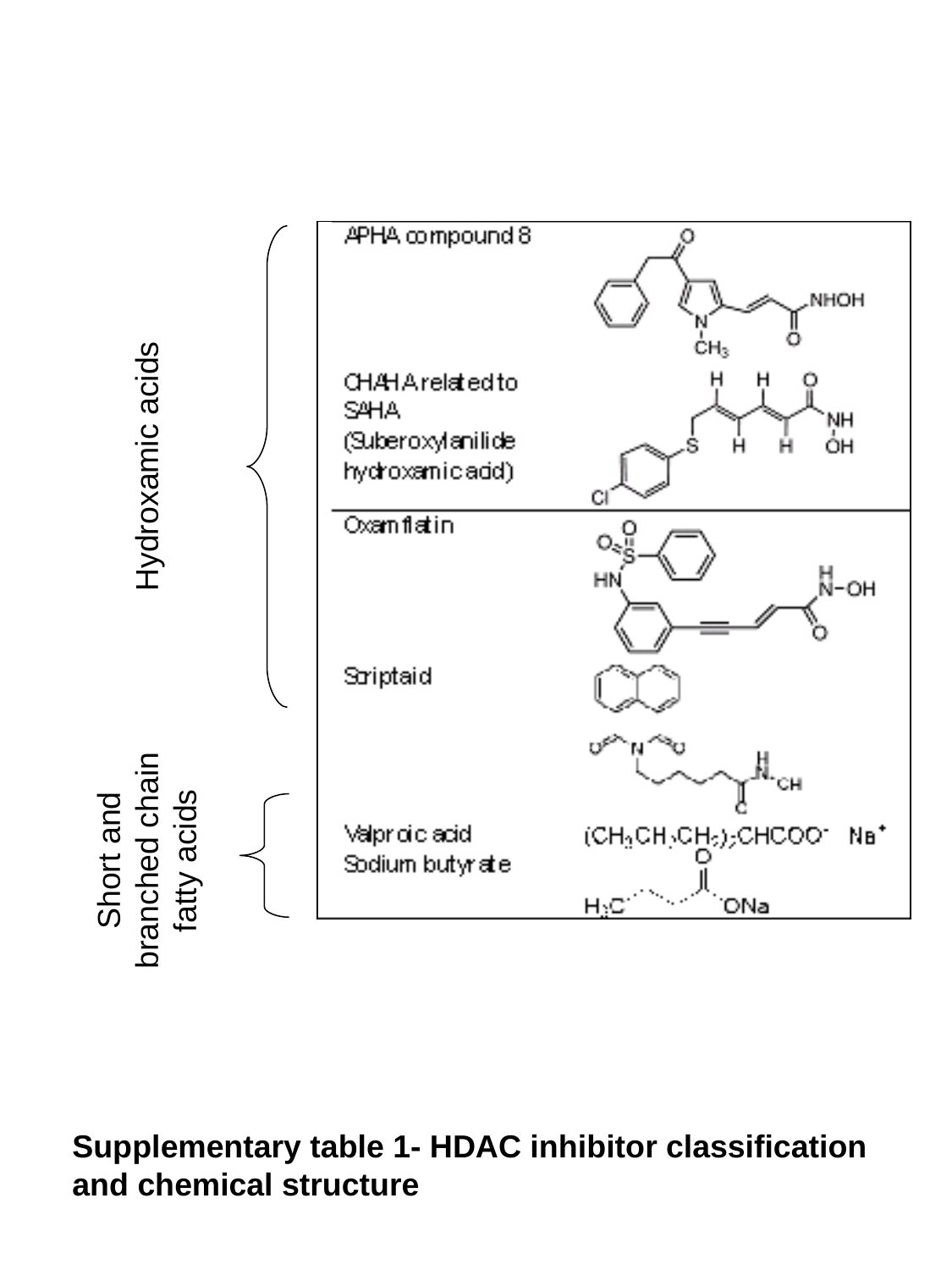

Hydroxamic acids
Short and branched chain fatty acids
Supplementary table 1- HDAC inhibitor classification and chemical structure

## Slide 3
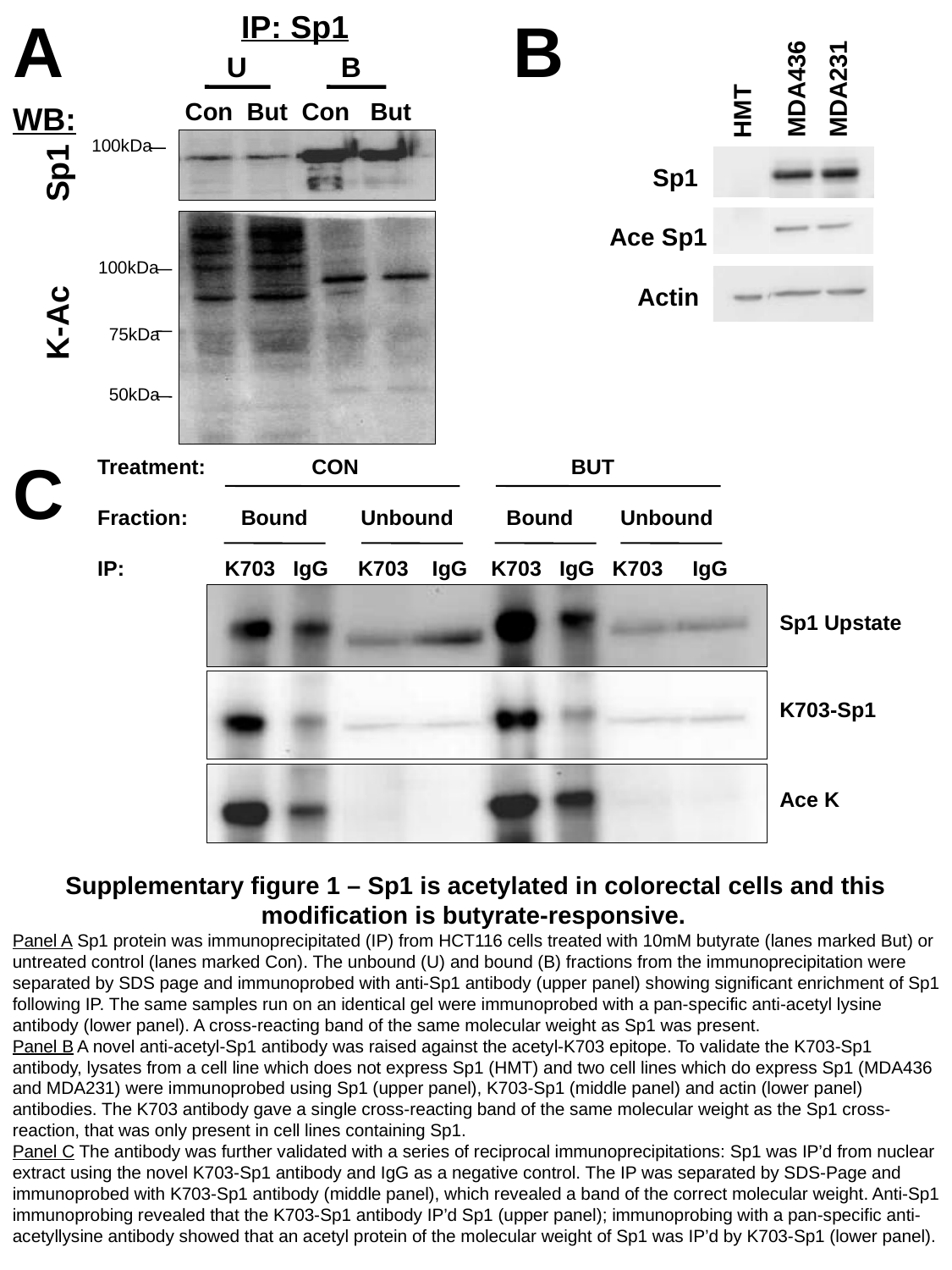

IP: Sp1
U
B
Con But Con But
WB:
100kDa
Sp1
100kDa
K-Ac
75kDa
50kDa
B
A
MDA436
MDA231
HMT
Sp1
Ace Sp1
Actin
C
Treatment: CON BUT
Fraction: Bound Unbound Bound Unbound
IP: K703 IgG K703 IgG K703 IgG K703 IgG
Sp1 Upstate
K703-Sp1
Ace K
Supplementary figure 1 – Sp1 is acetylated in colorectal cells and this modification is butyrate-responsive.
Panel A Sp1 protein was immunoprecipitated (IP) from HCT116 cells treated with 10mM butyrate (lanes marked But) or untreated control (lanes marked Con). The unbound (U) and bound (B) fractions from the immunoprecipitation were separated by SDS page and immunoprobed with anti-Sp1 antibody (upper panel) showing significant enrichment of Sp1 following IP. The same samples run on an identical gel were immunoprobed with a pan-specific anti-acetyl lysine antibody (lower panel). A cross-reacting band of the same molecular weight as Sp1 was present.
Panel B A novel anti-acetyl-Sp1 antibody was raised against the acetyl-K703 epitope. To validate the K703-Sp1 antibody, lysates from a cell line which does not express Sp1 (HMT) and two cell lines which do express Sp1 (MDA436 and MDA231) were immunoprobed using Sp1 (upper panel), K703-Sp1 (middle panel) and actin (lower panel) antibodies. The K703 antibody gave a single cross-reacting band of the same molecular weight as the Sp1 cross-reaction, that was only present in cell lines containing Sp1.
Panel C The antibody was further validated with a series of reciprocal immunoprecipitations: Sp1 was IP’d from nuclear extract using the novel K703-Sp1 antibody and IgG as a negative control. The IP was separated by SDS-Page and immunoprobed with K703-Sp1 antibody (middle panel), which revealed a band of the correct molecular weight. Anti-Sp1 immunoprobing revealed that the K703-Sp1 antibody IP’d Sp1 (upper panel); immunoprobing with a pan-specific anti-acetyllysine antibody showed that an acetyl protein of the molecular weight of Sp1 was IP’d by K703-Sp1 (lower panel).

## Slide 4
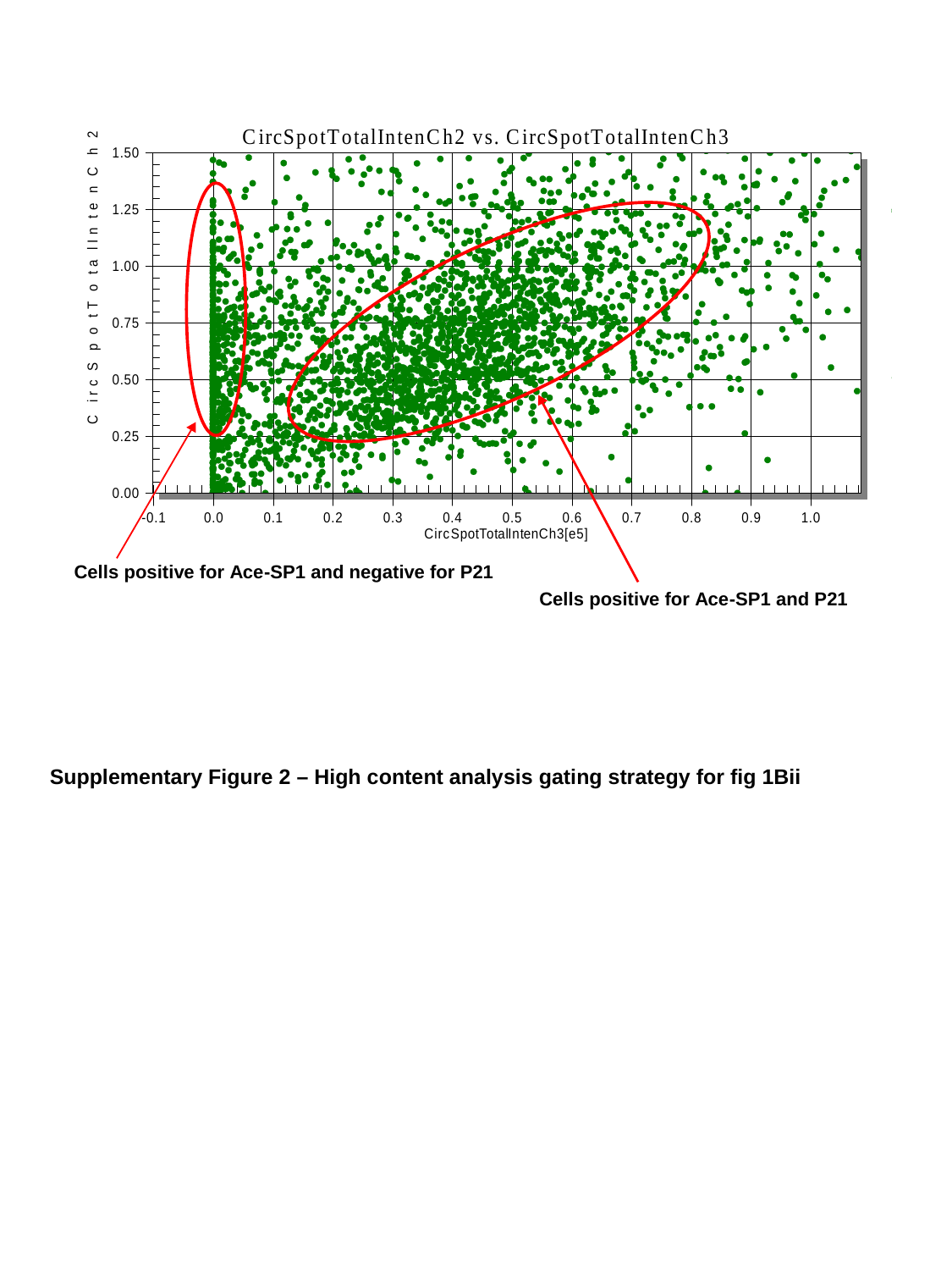

Supplementary Figure 2 – High content analysis gating strategy for fig 1Bii

## Slide 5
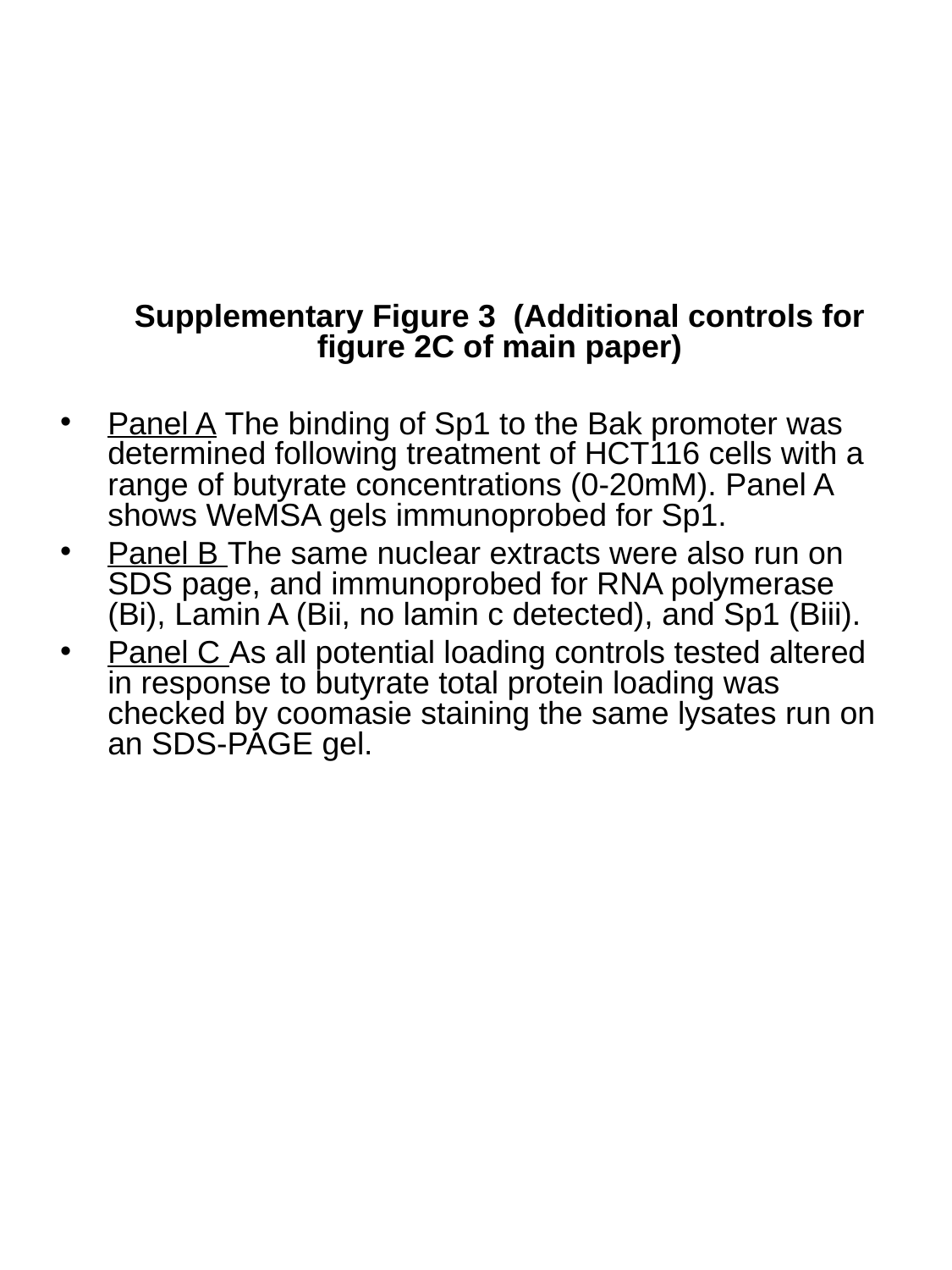

# Supplementary Figure 3 (Additional controls for figure 2C of main paper)
Panel A The binding of Sp1 to the Bak promoter was determined following treatment of HCT116 cells with a range of butyrate concentrations (0-20mM). Panel A shows WeMSA gels immunoprobed for Sp1.
Panel B The same nuclear extracts were also run on SDS page, and immunoprobed for RNA polymerase (Bi), Lamin A (Bii, no lamin c detected), and Sp1 (Biii).
Panel C As all potential loading controls tested altered in response to butyrate total protein loading was checked by coomasie staining the same lysates run on an SDS-PAGE gel.

## Slide 6
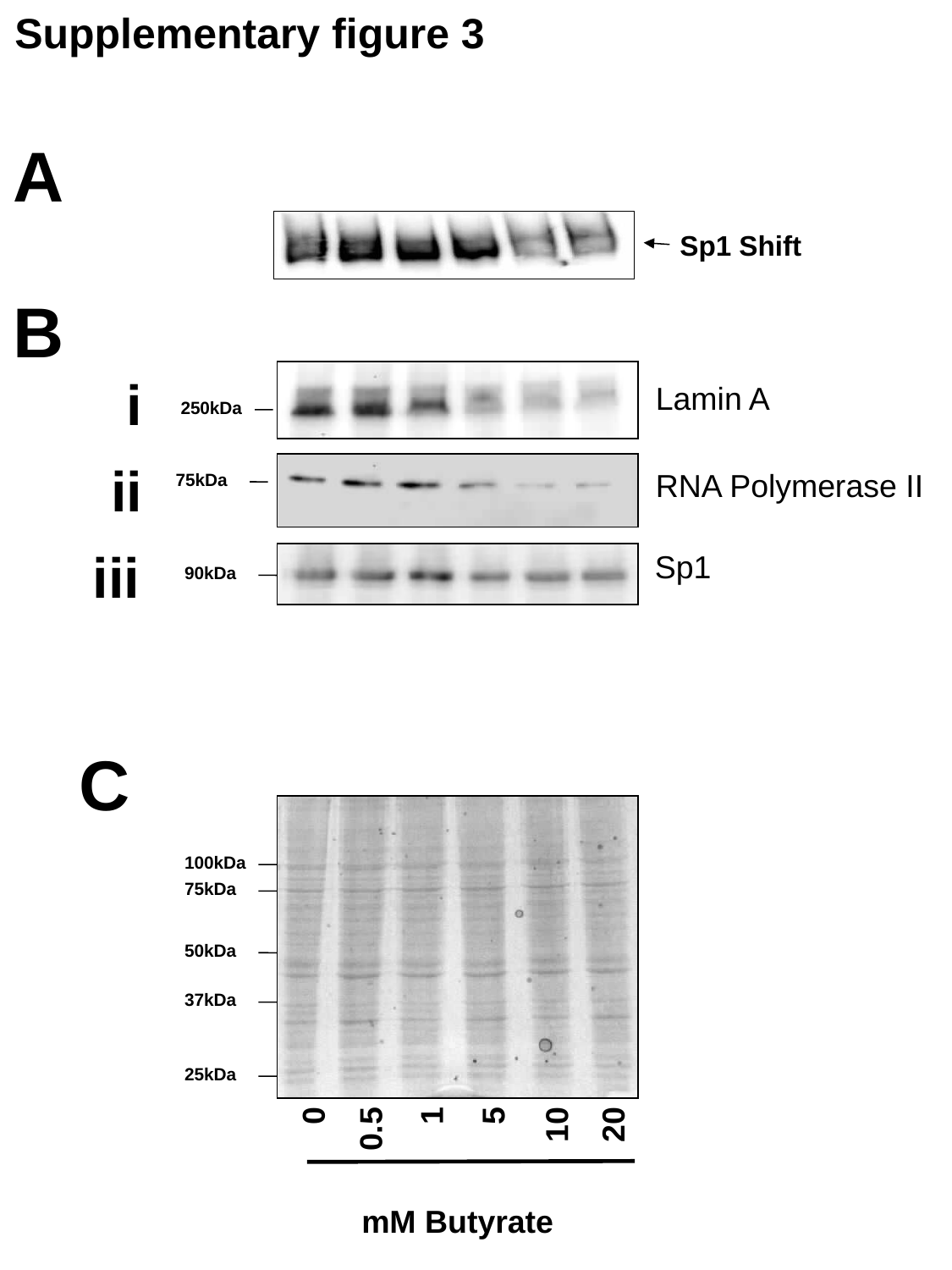

Supplementary figure 3
A
Sp1 Shift
B
i
Lamin A
250kDa
ii
RNA Polymerase II
75kDa
iii
Sp1
90kDa
C
100kDa
75kDa
50kDa
37kDa
25kDa
0
1
5
10
20
0.5
mM Butyrate

## Slide 7
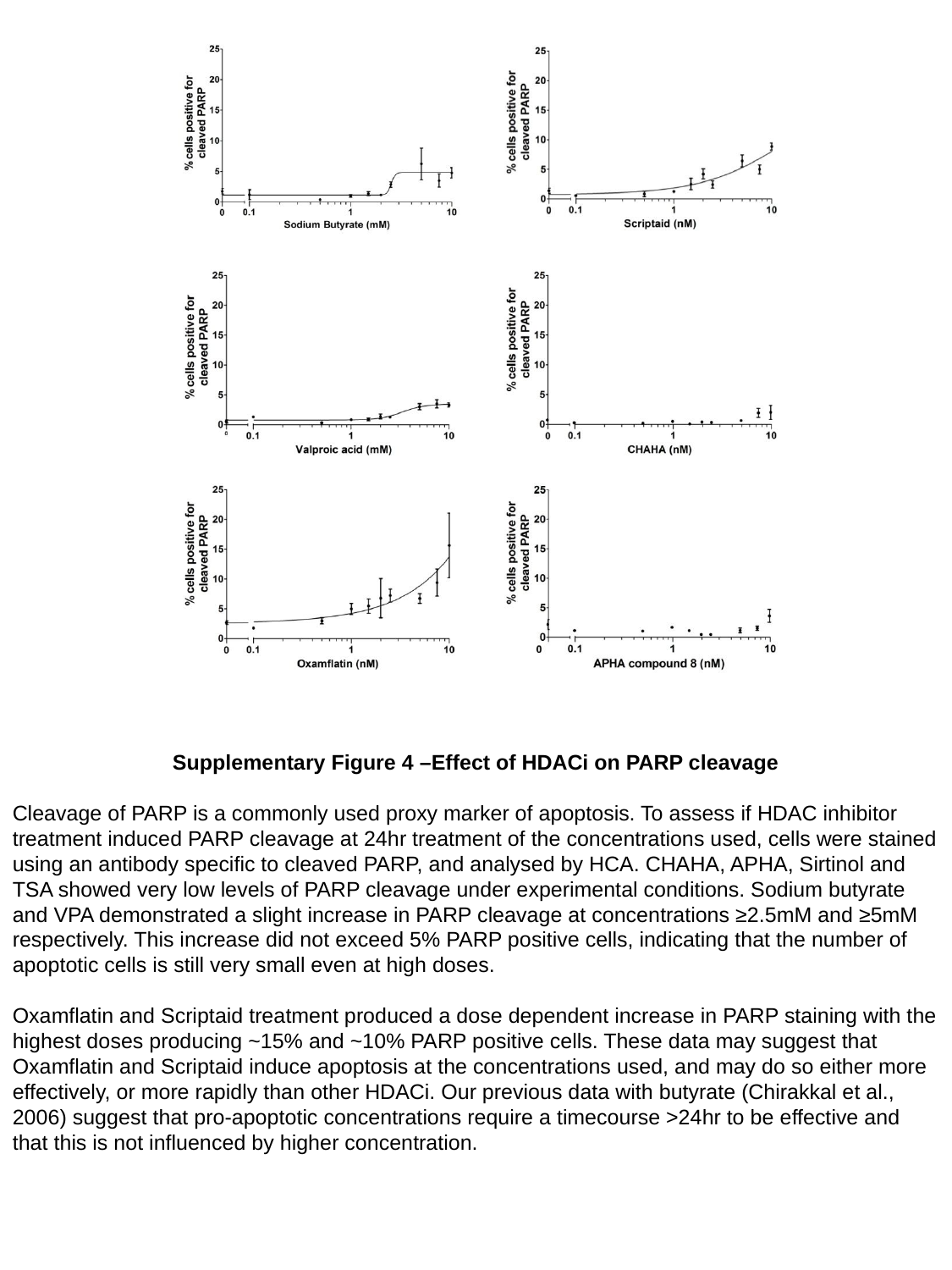

Supplementary Figure 4 –Effect of HDACi on PARP cleavage
Cleavage of PARP is a commonly used proxy marker of apoptosis. To assess if HDAC inhibitor treatment induced PARP cleavage at 24hr treatment of the concentrations used, cells were stained using an antibody specific to cleaved PARP, and analysed by HCA. CHAHA, APHA, Sirtinol and TSA showed very low levels of PARP cleavage under experimental conditions. Sodium butyrate and VPA demonstrated a slight increase in PARP cleavage at concentrations ≥2.5mM and ≥5mM respectively. This increase did not exceed 5% PARP positive cells, indicating that the number of apoptotic cells is still very small even at high doses.
Oxamflatin and Scriptaid treatment produced a dose dependent increase in PARP staining with the highest doses producing ~15% and ~10% PARP positive cells. These data may suggest that Oxamflatin and Scriptaid induce apoptosis at the concentrations used, and may do so either more effectively, or more rapidly than other HDACi. Our previous data with butyrate (Chirakkal et al., 2006) suggest that pro-apoptotic concentrations require a timecourse >24hr to be effective and that this is not influenced by higher concentration.
